# The Prevention of Venous Thromboembolism After Esophago-Gastric Surgery: A Never-Ending Story

**DOI:** 10.1245/s10434-022-11572-7

**Published:** 2022-03-29

**Authors:** Raffaele Rocco, Janani Reisenauer

**Affiliations:** Division of Thoracic Surgery, Department of Surgery, Mayo Clinic, 200 First Street SW, Rochester, MN 55905 USA

The study published in this issue of ASO by Hanna et al.^1^ reiterates a well-known dilemma in the peri- and postoperative management of patients with esophagogastric surgery.^2,3^ In fact, only a few other conditions may entail this prolonged risk of 2.5%.^1^ The literature has suggested that the risk for venous thromboembolism (VTE) (1.9%) may last for up to 2 years after complex gastrointestinal surgery.^3^ VTE can develop in single or recurrent episodes even after the traditional 90-day postsurgical period, thus mandating attention toward prolonged anticoagulation. Moreover, up to one third of the patients subjected to esophagogastric surgery may develop VTE after discharge. The message of this study is clear: prevention of VTE after esophagogastric surgery never ends. However, Nader and colleagues have uniquely searched for predisposing factors over a prolonged period of 6 months before and after the operation.

The authors of this interesting study also found that, while neoadjuvant chemotherapy was associated with VTE before surgery, extended hospital stay predicted VTE onset after surgery, being VTE itself is a cause of prolonged length of stay. Above all, Hanna et al.^1^ warn against the danger of ignoring the potential adverse effect of VTE on OS and CSS, which are severely reduced in affected patients (up to 50% with HR = 1.36 and 1.42, respectively).

Although the above results are not an absolute novelty, a few points need to be emphasized that add value to the literature. Almost 5,000 patients have been analyzed with an attempt at addressing a homogeneous cohort undergoing similar perioperative management from a regional database to capture demographic and clinical data. In this setting, the socioeconomic status of the study population has been added as an important covariate in the multifactorial analysis of perioperative VTE. The length of the study period is another strong point of this study, because 6-month intervals before and after surgery have been considered in the analysis. Moreover, patients with one or more episodes of VTE were included in the study to emphasize the need for prolonged surveillance and preventative measures. The relevance of the study by Nader et al. also is expressed by the fact that almost 60% of the patients in this cohort did not have major comorbidities when they underwent surgery; yet the reported incidence of VTE was 9% after esophageal and 7.5% after gastric surgery. The 30-day postoperative VTE incidence was 4.5% and 2.3%, after esophagectomy and gastrectomy, respectively, which compares favorably with the NESQIP data reported by De Martino et al. (7.3% and 2.7%, respectively) and from a recent review by Theochari et al. (4% after esophagectomy).^4,5^

Agzarian et al.^6^ showed that while 34% of surgeons estimated that more than 20% of VTE occurred post hospital discharge, only 13% routinely utilized extended thromboprophylaxis. The study by Nader et al. reiterates the importance of consensus based on evidence-based data.

The dilemma remains as to which factors can be modified to change the incidence of VTE after complex gastroesophageal surgery. As an example, the implementation of neoadjuvant treatment has been counteracted by the widespread adoption of enhanced recovery pathways, minimizing fluid overload, and early ambulation, which shorten postoperative hospital stay. This information is not included in the paper by Nader et al.

Final details that could be further investigated are the types of recommended anticoagulation as well as the implementation of scoring systems based on patient characteristics and biomarkers to predict risk.^5,7^

In conclusion, a prolonged anticoagulation prophylaxis seems to be an effective preventative measure against perioperative VTE after esophagogastric surgery. Still to be clarified will be the type of anticoagulation, whether mechanical preventative measures should be included, and most of all, how long this prophylaxis should last since the prevention of VTE after complex esophagogastric surgery appears to remain a never-ending story.^6,8^
